# Cost-utility of focused ultrasound compared to radiotherapy for Dutch patients with uncomplicated non-spinal bone metastases

**DOI:** 10.1007/s10198-025-01861-9

**Published:** 2025-12-05

**Authors:** Julia Simões Corrêa Galendi, Ingrid Nijholt, Renée Hovenier, Jorik Slotman, Erik Phernambucq, Stephanie Stock, Clemens Bos, Manon Braat, Helena Verkooijen, Martijn Boomsma, Dirk Müller

**Affiliations:** 1 Department of Internal Medicine, São Paulo State University/UNESP, Medical School, Sao Paulo, Brazil; 2 Institute of Health Economics and Clinical Epidemiology, University Hospital of Cologne, Cologne, Germany; 3 Department of Radiation Oncology, Isala Hospital, Zwolle, The Netherlands; 4 Division of Imaging and Oncology, University Medical Centre Utrecht, Utrecht, The Netherlands; 5 Department of Radiology, Isala Hospital, Zwolle, The Netherlands

**Keywords:** Bone metastases, MR-HIFU, Focused ultrasound, Cost-utility, Radiotherapy, Palliative care

## Abstract

**Introduction:**

Non-randomized clinical trial has shown that Magnetic Resonance guided High-intensity focused ultrasound (MR-HIFU) leads to faster pain relief compared to the current standard of care External Beam Radiotherapy (EBRT).

**Objective:**

To evaluate the cost-utility of ’early MR-HIFU’ (with optional EBRT afterwards) or ‘delayed MR-HIFU’ (i.e., MR-HIFU after failed EBRT) versus EBRT (with optional re-irradiation with EBRT) from the societal perspective in the Netherlands

**Methods:**

A lifelong patient-level simulation model was developed. After a treatment with either MR-HIFU or EBRT, a patient could have: (i) complete pain relief, (ii) partial pain relief, (iii) persistent pain and (iv) death. We also accounted for the event of a pathological fracture. Model outputs were costs, quality-adjusted life years (QALYs), and incremental cost-effectiveness ratios (ICER). The net monetary benefit was calculated considering the willingness-to-pay threshold of €80,000 per QALY gained, adjusted by the Dutch disease severity index. Deterministic and probabilistic sensitivity analyses were conducted.

**Results:**

The strategy ‘delayed MR-HIFU’ costs €706 more than EBRT, whilst ‘early MR-HIFU’ costs €1,875 more than EBRT.‘Early MR-HIFU’ adds 0,15 more QALYs than EBRT, resulting in an ICER of €12.755 per QALY and an incremental net monetary benefit of €8,631. At a threshold of 80,000€ per QALY there is a 77% probability that ‘early MR-HIFU’ is the most cost-effective option.

**Conclusion:**

Although there are still uncertainties relating to implementation of MR-HIFU in patient care, our modelling study shows that offering MR-HIFU as an early treatment would be the most cost-effective option in the Netherlands.

**Supplementary Information:**

The online version contains supplementary material available at 10.1007/s10198-025-01861-9.

## Introduction

Bone metastases present a substantial burden for cancer patients, causing debilitating pain and significantly impairing their quality of life [1]. The current standard of care for local treatment of painful bone metastases is external beam radiotherapy (EBRT), which results in effective pain relief in about 70% of patients [2, 3]. However, EBRT is associated with potential side effects, and pain decrease usually emerges not before four weeks post-treatment [2, 3].

High intensity focused ultrasound (HIFU) is a non-invasive treatment alternative that has the potential to cause pain relief within days after treatment and for a longer time than EBRT by ablating metastatic lesions [4]. For treatment planning and monitoring, HIFU can be performed under magnetic resonance imaging guidance (MR-HIFU), which allows safe and effective procedures with minimal damage to surrounding tissue [4]. In a placebo-randomized-controlled trial, 64% of patients treated with MR-HIFU reported pain relief (improvement in self-reported pain score) at 3 month-follow up, compared to 20% in the placebo arm (*p* <.001) [5]. More recently, a prospective non-randomized study comparing MR-HIFU and EBRT showed that pain relief at 1-month follow-up was 91% (91 of 100 patients) after focused ultrasound and 67% (66 of 98 patients) after EBRT (*p* <.001) [6]. Serious adverse effects with MR-HIFU are rare, and pain emerging from positioning oin the MRI table is usually resolved on the day of treatment [7].

Reimbursement is a key factor that might hinder the adoption of MR-HIFU in clinical practice in the Netherlands, as shown in an inquiry with multiple stakeholders [8]. In this context, an early cost-utility analysis can be useful for decision-makers for two main reasons. First, an early assessment of the potential cost-effectiveness of a medical device can inform early investment, aiding payers to avoid sunk costs of installation and personnel training which are usually associated with medical devices [9]. Second, early health technology assessments provide the opportunity to assess how alternative ways of offering innovative treatments within the care pathway may affect their cost-effectiveness [9].

The primary objective of this study was to assess the cost-utility of MR-HIFU compared to EBRT for selected patients with painful non-spinal bone metastases from a societal perspective in the Netherlands. A secondary objective was to investigate the impact of the order of MR-HIFU treatment in patient care (i.e., whether as a first-line treatment or as a second-line treatment) on the incremental cost-effectiveness ratio.

## Methods

To assess the lifetime economic and clinical consequences of adopting MR-HIFU for patients with bone metastases, a patient-simulation model was developed using TreeAge Pro 2024 (TreeAge Software, Williamstown, MA), and is reported according to the Consolidated Health Economic Evaluation Reporting Standards 2022 checklist [10]. According to the directive of the Dutch National Healthcare Institute (Zorginstituut Nederland -ZIN), the analyses were performed from the societal perspective and we applied differential discounting for costs (4%) and QALYs (1.5%) [11]. A lifetime horizon was chosen to reflect the impact of pain palliation in patients’ lifetime and the need for retreatment. The chosen cycle length was one month to reflect the time needed to observe a full treatment effect from EBRT or MR-HIFU.

The patient population comprised patients with breast, prostate or lung cancer with uncomplicated non-spinal bone metastases who undergo a preselection to assess eligibility for both EBRT and MR-HIFU [12]. According to the ESTRO-ACROP guidelines, bone metastases should be regarded as uncomplicated if they are (i) painful; (ii) without impending or existing pathologic fracture; and (iii) without spinal cord or cauda equina compression, irrespective of size or location [13]. Impending pathologic fracture is characterized as no immediate need of surgery or stabilization, assessed by a ≥ 9 points in the Mirels’ scoring system, which is recommended in the ESTRO-ACROP guideline [13].

Notably MR-HIFU is contraindicated in patients with spinal metastases and (impending) pathological fractures, and these patients are then excluded from our analysis [14]. The patient population was in accordance with the participants from the main study on MR-HIFU effectiveness, meaning patients with mean age of 64 years ± 13 (SD) [6].

### Strategies for the main comparison

**Fig. 1:**
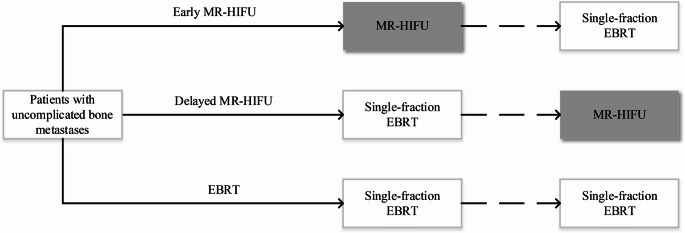
Strategies for the main comparison. Dashed lines indicate that not all patients would receive a second treatment, as patients might have successful pain palliation after first line therapy or might die before receiving a second treatment. EBRT = External Beam Radiotherapy, MR-HIFU = Magnetic resonance imaging High Intensity Focused Ultrasound

Three strategies were designed for the main comparison, all based on one first-line therapy and one salvage pain palliation therapy if needed. The salvage therapy is applied in case of treatment failure or if the patient later experiences pain recurrence after initially successful pain palliation (Fig. 1). The first strategy, henceforth called ‘early MR-HIFU’, was based on MR-HIFU as the first-line therapy, followed by EBRT if needed. The second, henceforth called ‘delayed MR-HIFU’ was based on EBRT as first line-therapy followed by MR-HIFU, if needed.

The third strategy reflected the current standard of care, i.e., EBRT followed by re-irradiation with EBRT. EBRT dose was defined as single fraction (8 Gy in one fraction). Because a previous study-based cost-utility analysis for the Netherlands concluded that multiple-fraction is not cost-effective compared to single-fraction [15], the multiple-fraction was not included in our model. In addition, more advanced radiotherapy techniques such as stereotactic radiotherapy (SBRT) were not considered as standard treatment for bone metastasis.

### Model structure

**Fig. 2:**
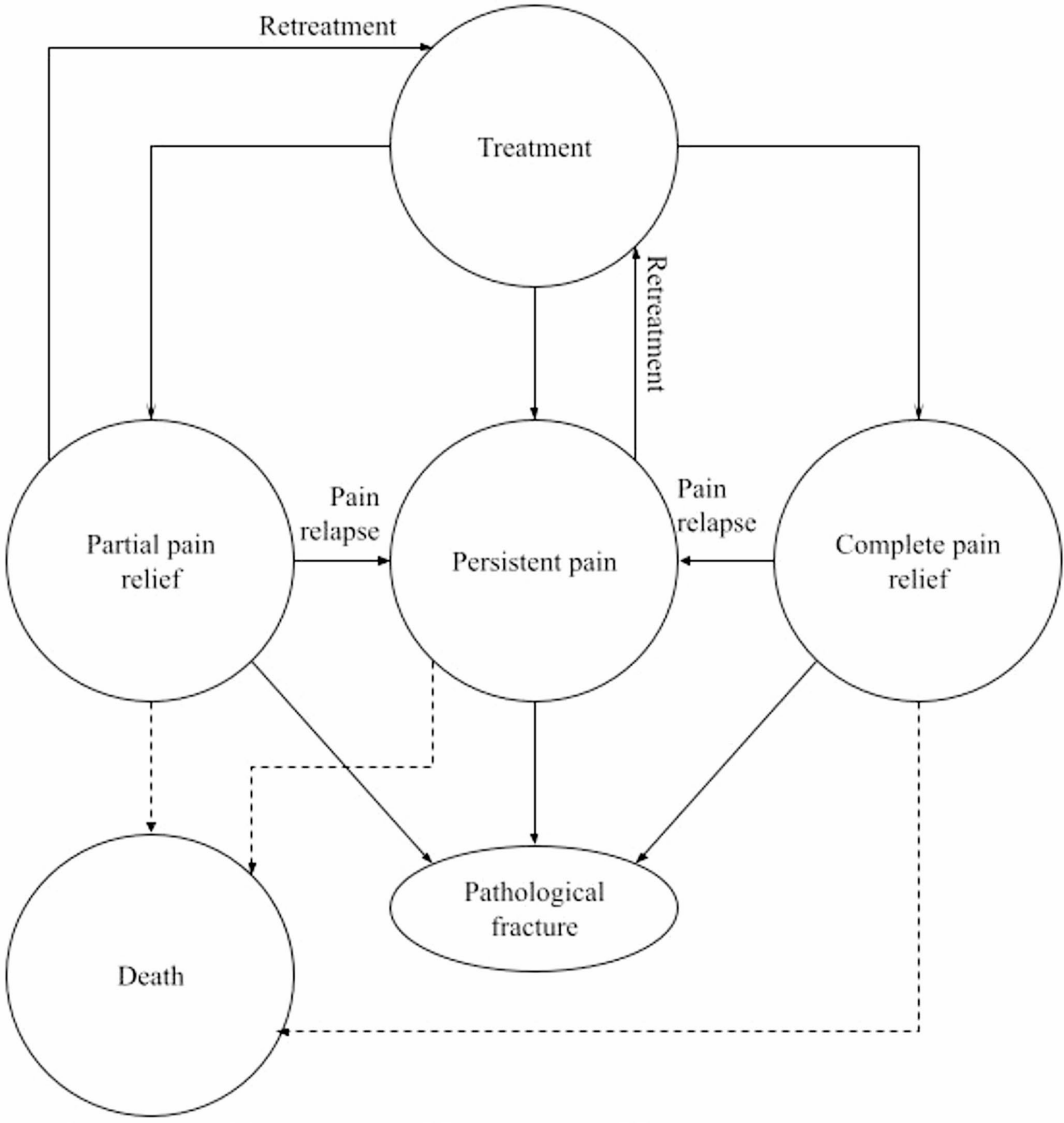
Model structure

The model structure was in line with that of a previous economic modeling study developed for Germany [16] (Fig. 2). After a treatment with either MR-HIFU or EBRT, a patient could have: (i) complete pain relief, defined as a pain score of zero on the numerical rate scale (NRS), (ii) partial pain relief, defined as reduction in pain score of at least two points without increase in analgesic intake, or (iii) persistent pain, and lastly (iv) death. Although the risk of pathological fractures is low in this patient population, fractures are costly and affect a patients’ quality of life [17]. Therefore, pathological fractures were considered to be events that could occur at any time, with patients suffering a pathological fracture progressing to (iii) persistent pain.

### Input parameters

To identify the appropriate input parameters, several literature searches in Medline (via PubMed) were undertaken. Details of the search strategies and main search results are provided in Online resource 1. Main input parameters applied to the model are presented in Table 1.

**Table 1:** Input parameters

Input parameters	Mean Value (SD)	Distributions	Source
MR-HIFU procedure	€ 5969 (1194)^a^	gamma	Calculated based on [22]
Single fraction EBRT	€ 2966 (593)	gamma	[15]
Oxycodone (cost per month)	€ 87,8	gamma	[30]
Dexamethasone (cost per day)	€ 0.34	gamma	[30]
Ondansetron (cost per mg/cost per three weeks)	€ 0.53/€ 134	gamma	[30]
Pathological fracture	€ 17,971	gamma	[25]
Travel costs (per visit)	€ 7.60	gamma	[24]
Average distance from home to hospital (excl. outpatient clinic away from a hospital)	7.1 km		[24]
Costs per kilometre	€ 0.26		[24]
Parking fees per visit	€ 3.92		[24]
Utilities	QALY Mean (SD)		
Bone metastases	0.47 (0.42)	beta	[19]
Fracture	− 0.36 (0.26)	lognormal	[19]
Single-fraction EBRT^b^	− 0.11 (0.31)	lognormal	[19]
MR-HIFU	− 0.05 (0.17)	lognormal	Assumption, [19]
Complete pain relief	− 0,23 (0,43)	lognormal	[20]
Partial pain relief	− 0,075 (0,22)	lognormal	[20]
Event probabilities			
HIFU			
Complete pain relief after	0.431 (0.048)	beta	[6]
Partial pain relief	0.480 (0.049)	beta	[6]
Risk of fracture (monthly probability)	0.003 (0.005)	beta	[5]
Pain relapse (monthly probability)	0,0087(0,003)	beta	[6]
Retreatment (monthly probability)	0.018 (0.0016^c^)	beta	Assumption, [2]
EBRT			
Complete pain relief	0.170 (0.037)	beta	[6]
Partial pain relief	0.510 (0.049)	beta	[6]
Fracture (monthly probability)	0.003 (0.0007)	beta	[2]
Pain relapse (monthly probability)	0.029 (0,002)	beta	[2]
Retreatment (monthly probability)	0.007 (0.0011)	beta	[2]
Monthly probability of death after bone metastasis diagnosis			
Breast cancer	1y: 0.040 (0.0004); 2y: 0.029; 3y: 0.029; 4y: 0.027; 5y: 0.027	beta	[37, 38]
Prostate cancer	1y: 0.053 (0.0018); 2y: 0.039; 3y: 0.034; 4y: 0.029; 5y: 0.028	beta	[38]
Lung cancer	1y: 0.070 (0.0005); 2y: 0.050; 3y: 0.050; 4y: 0.030; 5y: 0.020	beta	[38]

^a^ A standard deviation of 20% was assumed, ^b^ transitory decrease in quality of life due to adverse events and inconvenience of treatment ^c^ Assumed to be 20% higher than EBRT

y = year, EBRT = external beam radiation therapy, MR-HIFU = magnetic resonance image-guided high intensity focused ultrasound, SD = standard deviation

#### Event probabilities

Event probabilities for complete pain relief and partial pain relief were taken from a prospective non-randomized study comparing MR-HIFU and EBRT that included 198 patients for a 12-month follow-up [6]. For EBRT, we calculated the probability of relapses and risk of fracture considering only the single-fraction population from a systematic review with meta-analyses [2, 3]. After EBRT, 30% of patients have suffered a pain relapse at one year follow-up [3, 18]. For MR-HIFU, we considered that the probability of relapse would be lower, based on 12-month follow up data from a prospective open-label non-randomized phase II study [6].

#### Health state utilities

Quality-adjusted life years (QALY) values were obtained from two main sources. Based on time trade-off (TTO) interviews, 187 patients from the United Kingdom (UK) and Canada rated the impact of skeletal-related events on quality of life due to treatment/retreatment with either MR-HIFU or EBRT [19].

Based on EQ-5D-5 L data for patients with chronic pain [20], for patients with complete and partial pain relief, after treatment an increase in utility of 50% and 15%, respectively, was assumed. This assumption was also used in previous economic modeling studies [16, 21].

#### Costs

The costs of MR-HIFU and EBRT treatments included the resource use of pre-treatment imaging. Costs of MR-HIFU procedure were calculated using a micro costing approach [22], using costs from the ZIN reference list, and salary scales from CAO hospital sector (for salaried service staff) and medical staff (AMS) (for medical staff) [23, 24]. The micro costing approach is described in detail in Online resource 2. Costs of EBRT were taken from a Dutch trial-based cost-utility study [15]. The costs of treating a non-vertebral fracture were in accordance with a previous Dutch cost-utility analysis of acid zoledronic [25]. Costs were adjusted for inflation using the consumer price index [26].

We included travel costs (i.e., transport costs for outpatient care) and medication costs (e.g., analgesic, antiemetic, dexamethasone). According to the ZIN costing manual a kilometer price of a car was assumed as a proxy because that is the means of transport most commonly used to travel to a care institution [24].

After MR-HIFU, all patients were assumed to receive dexamethasone for three days to prevent flare-ups, based on practices in one of the selected centers. After EBRT, 4% of patients received dexamethasone for five days only if indicated [27]. Oxycodone was used as proxy medication to reflect analgesic use, an assumption previously done by similar modelling studies [16, 21, 28]. A dose of 20 mg every four hours was assumed for persistent pain and partial pain relief. Lastly, we accounted for costs of antiemetic medication in a proportion of patients with radiotherapy-induced nausea and vomiting (i.e., 50%). These patients were assumed to be taking daily 8 to 16 mg of ondansetron for three weeks [29]. Prices of medication were taken from the ZIN reference lists [30].

Although a societal perspective was taken, productivity losses arising from patients’ inability to work and due to premature death were not included, because patients enter the model at the age of 64 and bone metastases represent an advanced-stage disease with high mortality. Since there is no evidence that pain relief reduces the need for mobility aid, costs of formal and informal care were assumed to be the same in both groups and were excluded.

### Model output and validation

To validate the model, experts were invited to check the appropriateness of the model concept and input data (i.e., face validation). In addition, cross validation was performed by comparing the assumptions applied in our model to those of similar models [16, 21], and by comparing the model outputs with the results of other modeling studies [16, 21] and trial-based cost-utility studies [15]. Relevant literature was identified to validate the assumptions regarding utility values and pain recurrence, as described in Online Resource 3 [31–33]. The validation efforts followed the ‘Assessment of the validation Status of health Economic Decision Models’ (AdViSHE) checklist and are described in detail in Online resource 3 [34].

Model outputs were lifelong costs, QALYs, and incremental cost-effectiveness ratios (ICER). The net monetary benefit (NMB) was calculated considering the willingness-to-pay (WTP) threshold of €80 000 per QALY gained. The WTP threshold was determined by calculating the disease severity index for bone metastases [11]. A detailed description of the condition and the calculation of the disease severity index is presented in Online resource 4. In addition, we calculated the incremental NMB which indicates the difference in NMB between alternative interventions, with a positive incremental NMB indicating that the intervention is cost-effective compared to the next cheapest alternative [35].

### Sensitivity analyses and subgroup analyses

In deterministic sensitivity analyses (DSA), parameters were varied one by one to assess the effect on the ICER. The parameters tested in DSA were: the response rates and fracture risk for MR-HIFU, the probability of pain relapse after MR-HIFU, all utility values, and the costs of MR-HIFU, EBRT and treatment of pathological fractures, respectively. Costs of MR-HIFU were varied according to the best- and worst-case scenario resulting from our micro costing calculation.

A probabilistic sensitivity analysis (PSA) was performed to test overall uncertainty of the model results. In the PSA, the model was run 10,000 times with all parameters varied simultaneously within a priori defined distributions. Probabilities and utilities were assigned a beta distribution and costs a gamma distribution. Increments and decrements of utility values were assigned a lognormal distribution [36].

Mortality is expected to differ among different metastatic cancers depending on the primary disease. To explore the impact of the patient heterogeneity in model results, subgroup analyses were conducted for the three patient subgroups: metastatic breast cancer, metastatic prostate cancer and metastatic lung cancer.

## Results

### Base case

Over the patients’ lifetime, EBRT was the cheapest strategy (€6,508), and ‘early MR-HIFU’ was the most expensive strategy (€8,383). Table 2 shows the three strategies ranked from the less costly to the more expensive, and the incremental costs and QALYs in comparison with the EBRT strategy.

**Table 2:** Base case results

Strategy	Cost (€)	Incr. Cost (€)	Effectiveness (QALY)	Incr. Effectiveness (QALY)	ICER (€/QALY)	NMB (€)	INMB (€)
EBRT	6,508	reference	1.04	reference	reference	76,364	reference
Delayed MR-HIFU	7,214	706	1.06	0.02	28.816 (extendedly dominated)	77,618	1,254 (extendedly dominated)
Early MR-HIFU	8,383	1,875	1.18	0.15	12.755	86,249	8,631

*QALY* quality-adjusted life years, *ICER* incremental cost-effectiveness ratio, *NMB* net monetary benefit (calculated for the 80,000 willingness-to-pay threshold), *INMB* incremental net monetary benefit, *SF-EBRT* single fraction external beam radiotherapy, *MR-HIFU* magnetic resonance high intensity focused ultrasound

The strategy ‘delayed MR-HIFU’ costs €706 more than EBRT, whilst ‘early MR-HIFU’ costs €1,875 more than EBRT. Both MR-HIFU strategies generated slightly more QALYs than EBRT: ‘delayed MR-HIFU’ generates 0,02 more QALYs than EBRT, and ‘early MR-HIFU’ generates 0,15 QALYs more than EBRT (resulting in an extendedly dominated ‘delayed MR-HIFU’). Results for metastatic breast, prostate and lung cancer subgroups were consistent to the base case analyses and are shown in Online Resource 5.

### Results of sensitivity analyses

#### Deterministic sensitivity analyses

**Fig. 3:**
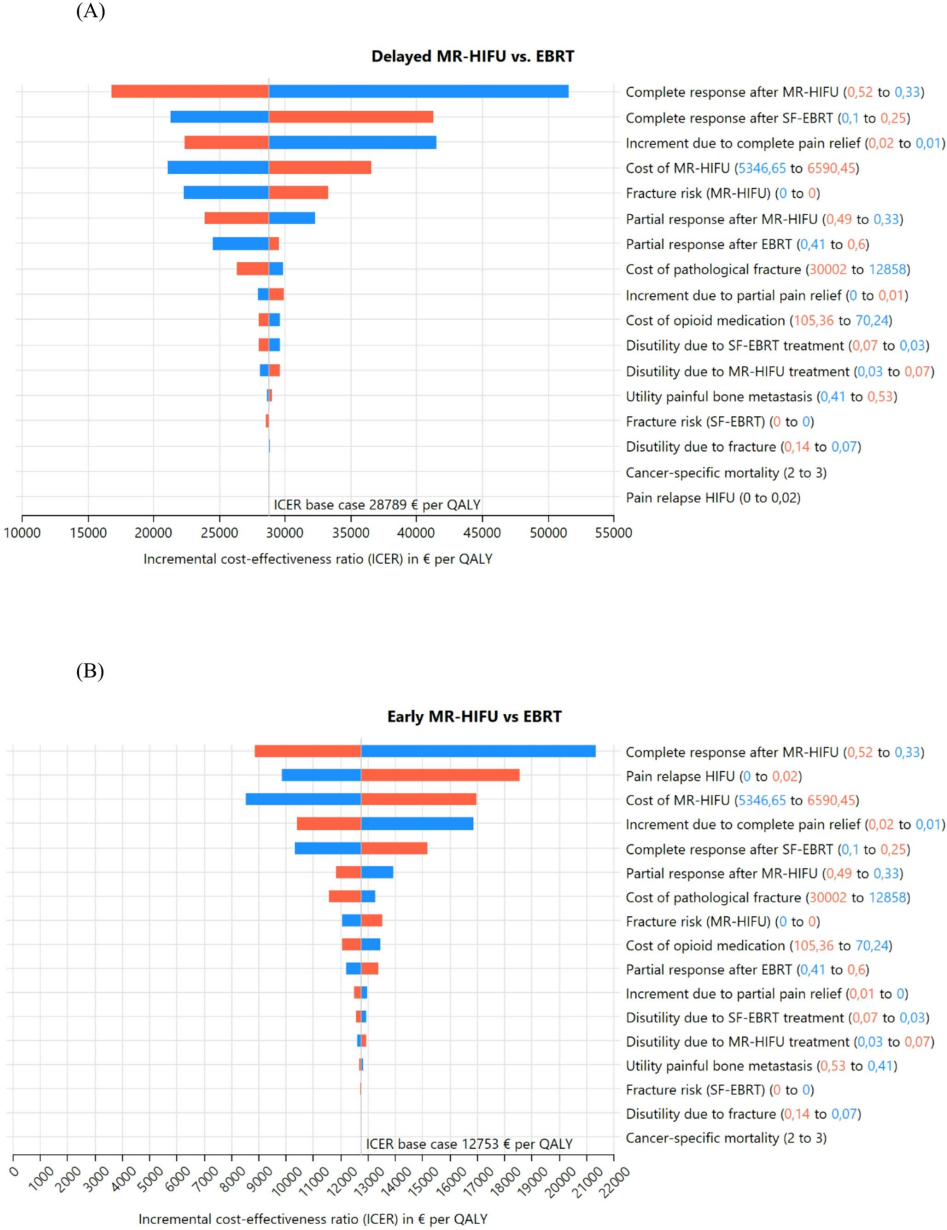
Tornado diagrams show the results of deterministic sensitivity analysis for the comparisons: (**A**) ‘Delayed MR-HIFU’ and EBRT and (**B**) ‘Early MR-HIFU’ vs. EBRT. Red bars represent lower bound parameters and blue bars represent upper bound parameters. EBRT: single fraction external beam radiotherapy, MR-HIFU: magnetic resonance high intensity focused ultrasound

Four parameters strongly affected the cost-effectiveness of ‘delayed MR-HIFU’ compared to EBRT: the probability of complete response after an MR-HIFU treatment, the probability of complete response after an EBRT, the increment in utility for complete pain relief and the cost of MR-HIFU (Fig. 3A). When comparing ‘early MR-HIFU’ and EBRT, the probability of complete response after MR-HIFU, the probability of pain relapse after an MR-HIFU treatment, the cost of MR-HIFU are the parameters that mostly affected the ICER (Fig. 3B).

#### Probabilistic sensitivity analyses

**Fig. 4:**
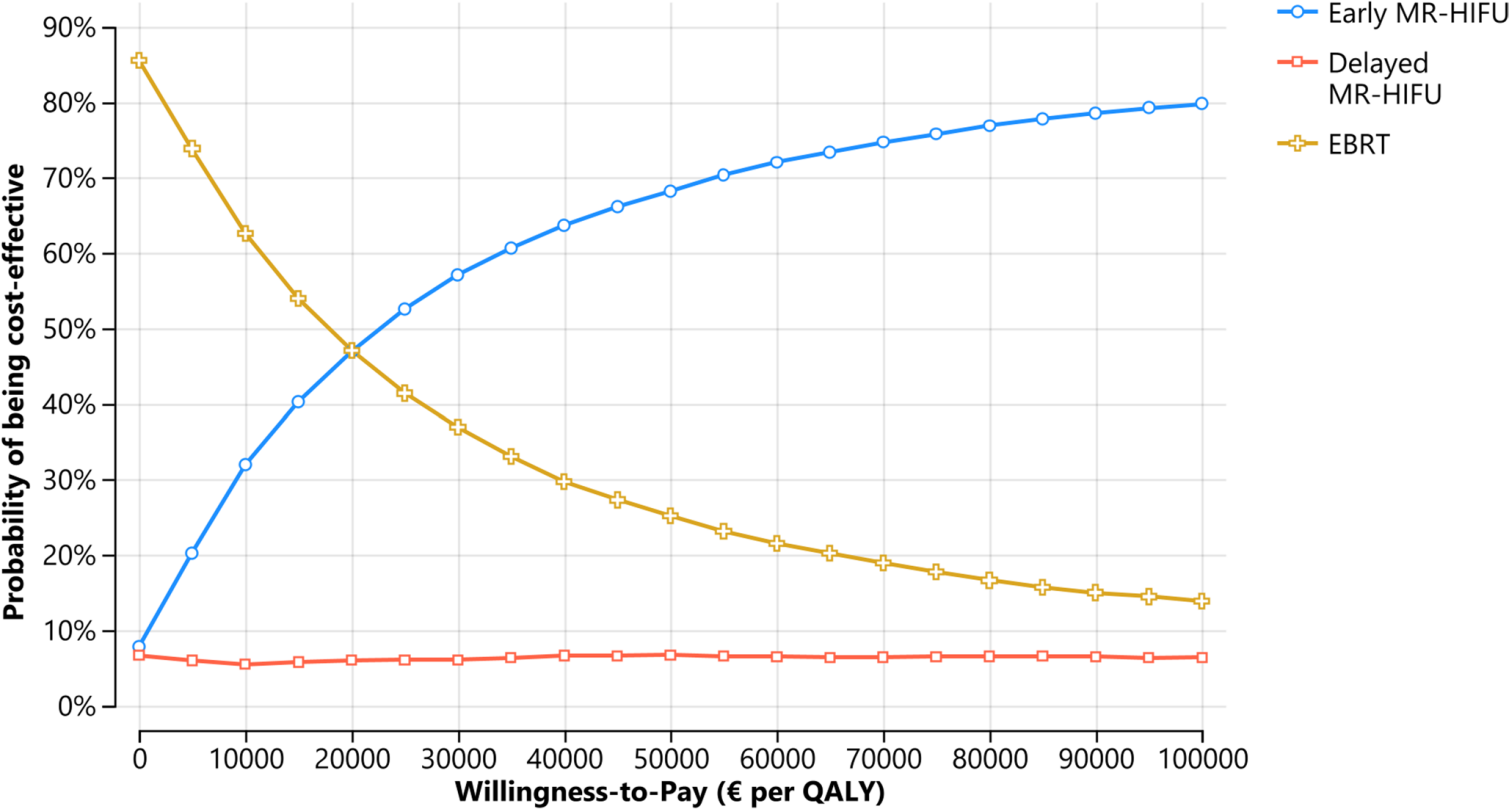
Cost-effectiveness acceptability curve. EBRT: single fraction external beam radiotherapy, MR-HIFU: magnetic resonance high intensity focused ultrasound

Above a willingness to pay-threshold of 80,000 € per QALY the probability of cost-effectiveness of ‘early MR-HIFU’ is higher than that of EBRT. At a threshold of 80,000€ per QALY there is a 77% probability that ‘early MR-HIFU’ is the most cost-effective option, as shown in Fig. 4 Scatterplots showing the PSA results in the incremental cost-effectiveness plane for the comparisons are provided in Online resource 6.

## Discussion

 In this study, the cost-utility of early and delayed MR-HIFU was compared with current standard care, i.e. single fraction EBRT, for patients with uncomplicated bone metastases from a societal perspective in the Netherlands. In comparison to EBRT, both MR-HIFU-based strategies generated more QALYs than EBRT over a patient’s lifetime at a moderately higher cost. In subgroup analyses, results were similar for the three cancer types considered in the analysis. Offering MR-HIFU early in the patient care pathway (i.e., as a first line treatment) more than doubles the QALY gain compared to offering MR-HIFU for patients only after failed EBRT (delayed MR-HIFU). Compared to EBRT, the early MR-HIFU strategy is cost-effective with an ICER of €12,755 per QALY.

 To reflect the societal perspective, we included travel costs in the analysis, considering the recommended input parameters from the ZIN methodological guideline. However, for EBRT and MR-HIFU, the average distance from home to hospital might be higher than that considered in our analysis. Moreover, costs of productivity losses and premature death were excluded due to the high mortality in this patient group, although survivorship after bone metastasis is increasing due to new treatment modalities [39]. For reference, the patient population that received MR-HIFU in the study form Napoli et al., had a mean survival of 5 to 7 months [6].

 It is important to highlight that not all patients with painful bone metastases will be eligible to MR-HIFU [12]. For instance, patients with spinal metastases or with pathological fractures needing surgical stabilization were excluded from clinical studies with MR-HIFU [5, 6]. In addition, patient selection for MR-HIFU depends on the clinical judgement of treating physicians, who will weigh factors such as the ability to reach the lesion with MR-HIFU and if there is soft tissue or nerve involvement [14]. In addition, referral to MR-HIFU could be impacted by the judgement from the referring physician, that might consider patient condition and comorbidities. Previous interrater agreement study indicates that there is a high need for objective and multidisciplinary eligibility criteria for referring patients to MR-HIFU [12]. A prospective observational study identified that 57 out of 741 (8%) of non-spinal bone lesions were eligible to MR-HIFU as alternative to EBRT, accounting for real-world limitations in patient eligibility for MR-HIFU [40].

 The main strength of the analysis is the flexible model structure that included different states of pain, i.e., persistent pain, partial pain and complete pain relief with the option of pain relapse and requirement of retreatment from each of these states. Thus, the model reflects the dynamic nature of pain palliation in painful bone metastases. The model was based on a previous economic modeling study developed for Germany [16], with both conceptualization and parametrization of the model conducted in agreement with clinicians and radiologists.

 A further strength of this modelling study is that beforehand the costs of MR-HIFU were calculated using a micro costing approach which identified ‘time’ as main cost-driver [22]. Based on a time-driven activity-based costing approach (TD-ABC), a European care-pathway (including a macro-, meso-, and micro-level) was designed to incorporate the care-delivery value chain. In recent years, TD-ABC has most often been used to calculate costs of inpatient procedures, to identify improvement opportunities in the workflow, or to support value-based initiatives [41]

Considering the micro costing study (Online resource 2), the total costs could be reduced to €4,000 or less by addressing two key cost-driving factors. First, by making MR-HIFU treatments more agile, which could be achieved by making improvements to the care pathway that reduces MRI room occupancy. Second, by increasing the operational capacity of the HIFU equipment to 16 h-per-week (instead of the current average 8 h-per-week) would result in a more favorable capital asset utilization, thereby reducing the total cost of MR-HIFU to €4,000 or less.

Retreatment following initial palliative radiotherapy is common in clinical practice [42]. However, retreatment rates are higher after single fraction treatments than after multifractionated regimens, assumedly because physicians are more willing to retreat considering the lower radiation dose given in single-fraction than in multifaction regimens [42]. There are direct implications for the overall economic evaluation of EBRT approaches since multifractionated schemes are costlier [15, 42].

The potential cost-effectiveness of MR-HIFU in comparison to placebo has been demonstrated in the United States setting [21]. In addition, a previous patient-level simulation model showed that MR-HIFU in comparison to EBRT is potentially cost-effective from the payer perspective in Germany, with an incremental cost-effectiveness ratio (ICER) of €19,845/QALY. However, according to national guidance for treatment, in the German model, only 10% of patients in the EBRT group received single fraction dose, while 90% received multi-fraction dose [16]. In contrast, in the Netherlands since single fraction is well established as the preferred option when radiotherapy is offered [15].

Some limitations have to be acknowledged. First, due to a lack of strong evidence, for several parameters the model had to count on various assumptions, resulting in some uncertainty in the results (i.e., effectiveness of MR-HIFU and cost of MR-HIFU). However, in the deterministic sensitivity analyses, for all variations the ICER remained below € 25,000 per QALY. With regard to the lack of high-quality data on efficacy of MR-HIFU, an ongoing RCT comparing MR-HIFU and EBRT is currently recruiting patients with bone metastases in four European countries (ClinicalTrials.gov registration number NCT04307914) [14].

Second, because there was no data on utilities and pain recurrence rates for bone metastases patients elicited from the Dutch population, we had to rely on international data [19]. However, the utility data was validated against the utilities directly measured in the cost-utility analysis from the Dutch datasets [15]. In the Dutch Bone Trial which assessed cost-utility of single-fraction versus multi-fraction radiotherapy mean total utility was about 0.4 (similar to the input of our model) [15]. Moreover, varying utilities in DSA affected the ICER only slightly. Regarding pain recurrence rates, a model validation against Dutch cohort lends credibility to our assumption of 20% probability for pain relapse after 1 year. The model input parameters data from the Dutch Bone Trial show that patients who respond to radiotherapy experience pain relief for approximately half of their remaining life with observed reirradiation due to pain relapse after single fraction EBRT of 26% [43].

Third, the cost data of EBRT treatments and fractures (although adjusted for inflation) could be outdated with regard to changes of treatment patterns. To account for this, we tested variations in these costs in sensitivity analysis. Assuming fractures as a homogenous event, the costs of treatment for a pathological fracture were less likely to impact on the results in sensitivity analysis [44]. However, the ICER was sensitive to the costs of EBRT. These costs had to be taken from a previous economic evaluation because more recent data on EBRT costs were not available. The feasibility of measuring EBRT costs with the TD-ABC method has been shown in international studies [45, 46], and could be a valid alternative for future studies attempting to update the estimation of EBRT costs in the Netherlands.

Lastly, we assumed that all patients would receive dexamethasone before MR-HIFU treatments, as per practice of one of the centers consulted. During model validation a second MR-HIFU center reported that dexamethasone prescription is not standard practice, and instead, patients might increase analgesic intake in the day after procedure due to pain flare-ups. However, the impact of this assumption is expected to be negligible, given that the costs of opioid medications on the ICER was very low in deterministic sensitivity analysis.

In conclusion, MR-HIFU-based strategies generated slightly more QALYs over a patient’s lifetime at higher cost compared to EBRT from a societal perspective in the Netherlands. Therefore, ‘early MR-HIFU’ could be a cost-effective treatment option. Although there is some uncertainty in the results, our analysis can aid payers and care providers to strategically plan the implementation of MR-HIFU in patient care.

## Supplementary Information

Below is the link to the electronic supplementary material.

Supplementary Material 1 (DOCX. 27.5 KB)

Supplementary Material 2 (DOCX. 605 KB )

## Data Availability

All research data for this work will be made available upon reasonable request to the corresponding author.
