# Supplementary material for: Cost-utility of focused ultrasound compared to radiotherapy for Dutch patients with uncomplicated non-spinal bone metastases
Source: Eur J Health Econ. 2025 Dec 5;27(5):1061–72. doi: 10.1007/s10198-025-01861-9 (PMC13451289; doi:10.1007/s10198-025-01861-9)
Supplement: Supplementary file 2 [file 10198_2025_1861_MOESM2_ESM.docx]

Cost-utility of focused ultrasound compared to radiotherapy for Dutch patients with uncomplicated bone metastases

Contents

[Online resource 1 – Literature search	2](#_Toc194658194)

[Online resource 2 – Micro costing of MR-HIFU	3](#_Toc194658195)

[Online resource 3 – Model Validation	6](#_Toc194658196)

[Online resource 4 - Calculation of disease severity	9](#_Toc194658197)

[Online resource 5 – Base case results and subgroup analyses	11](#_Toc194658198)

[Online resource 6- Scatterplots of probabilistic sensitivity analysis results	12](#_Toc194658199)

# Online resource 1 – Literature search

Search strategies:

((("Bone metastases"[Title/Abstract]) OR ("cancer pain"[Title/Abstract])) OR ("skeletal-related events"[Title/Abstract])) AND ((((("standard gamble"[Title/Abstract])) OR ("time trade off"[Title/Abstract])) OR ("health-related quality of life"[Title/Abstract])) OR ("health state utility"[Title/Abstract])). Ran on 02/02/23: 151 results

# Online resource 2 – Micro costing of MR-HIFU

Costs of MR-HIFU procedure were calculated based on a European cost-accounting framework using the time-driven activity-based costing (TDABC) approach (Simões Corrêa Galendi, 2022). A care pathway was designed based on input from European centers, including two Dutch Centers (UMCU and ISALA), and comprise mainly eight modules: outpatient consultation, pretreatment imaging, preparation, optimization, sonication, post treatment recovery and anesthesia.

Outpatient consultation referred to the appointment with the interventional radiologist for medical assessment and informed consent. Pre-treatment imaging consist of any imaging needed before treatment day, and may involve MRI or computerized tomography, according to the case requirements and depending on the availability of images from the ongoing oncologic follow up. The MR-HIFU treatment was separated in three modules: (i) the preparation module contains all activities for preparing the patient and the MRI room (e.g., dressing for treatment, quality assurance test); (ii) the optimization module starts with positioning the patient at the HIFU table, includes the planning MRI, and finishes with the test sonication; (iii) the sonication module refers to the therapeutic sonications and respective cooling time. The post-treatment module includes one control MRI and cleaning the MRI room. Lastly, the recovery module includes a stay at the recovery room and overnight at the clinical ward.

Resource consumption (e.g., room occupancy, personnel time, and equipment usage) were estimated by physicians, technicians, and clinical researchers from both university hospitals.

Then, resource consumption was combined with unitary cost data, mostly retrieved from the reference price list from the ZIN guidelines. All costs were adjusted for inflation based on the consumer price index 2024 [1].

Table S1. Resource consumption per module of the pathway.

| Module | Resource consumption description | Probability of resource use (%) | Time estimation (min) |
| --- | --- | --- | --- |
| Outpatient consultation | - | 1 | 30 |
| Pretreatment Image | MRI | 0.5 | 30 |
| Preparation | - | 1 | 77 |
| Optimization | - | 1 | 60 |
| Sonication | - | 1 | 90 (60-120)* |
| Post treatment | - | 1 | 30 |
| Recovery | - | 1 | 180 |
| Anesthesia | General anesthesia | 0.2 | 110 (110-170)* |
|  | Sedation | 0.8 | 110 (110-170)* |

*Ranges tested in best- and worst-case scenarios

Personnel costs

To calculate personnel costs, we considered 2024 price level salaries average salary paid by 1 full-time equivalent. As recommended in the 2024 ZIN guidelines, salary scales were taken from CAO hospital sector (for salaried service staff) and were based on salary scales salaried medical staff (AMS) (for medical staff). For University hospitals, salary scales were taken from CAO UMC. The standard working hours are 1536 hour per year, and for medical staff 2087 hours per year [2]. In the recovery room, we considered one anesthesiologist and one nurse for each four beds.

Table S2. Capacity cost rate (in € per min) for personnel.

|  | Capacity cost rate (€ per min) |
| --- | --- |
| Technician | 0.90 |
| Nurse | 0.75 |
| Interventional radiologist | 1.60 |
| Anesthesiologist | 1.60 |
| Sedationist | 0.90 |

Equipment costs

The cost of HIFU device is based on tender. We assumed that the HIFU device is used 320 hours per year, considering that the HIFU device is used for bone metastases and uterine fibroids. The costs of MRI and CT were calculated considering the 2024 reference prices from the ZIN guideline and a duration of 10 minutes for a CT and 30 minutes for an MRI.

Table S3. Capacity cost rate equipment.

| Equipment | Capacity cost rate (€ per min) | Reference prices (€) |
| --- | --- | --- |
| HIFU | 5.95 |  |
| MRI | 8.90 | 267.00 |
| CT | 18.80 | 188.00 |

Operating costs and disposables

Operating costs for a clinical ward and outpatient consultation were taken from the reference price list (Ref ZIN guideline). To calculate operating costs of the radiology department, cost/m2/year were provided by the hospital (i.e., maintenance, energy, water, heating/cooling of the MRI space). To calculate the Cost/m2/min we considered the dimensions of a regular MRI facility according to the International Health Facility Guidelines (Ref). These are: 64 m2 for the MRI room (Scanning room, computer equipment and control room), 35 m2 for preparation (anesthetic induction room, viewing and reporting room, change cubicle). Unitary costs of disposables were based on purchase prices provided by the hospitals. We considered 80% of patients would need a gel pad.

Table S4. Operating costs and disposables.

| Operating Costs | Capacity cost rate (€ per min) |
| --- | --- |
| Clinical Ward | 0.45 |
| Outpatient Consultation | 1.00 |
| MRI | 2.67 |
| Disposables | Unitary Cost (€) |
| Ultrasound gel | 13.49 |
| Gel Pad | 195.36 |
| Contrast - dotarem 15 ml | 183.63 |
| Contrast - optistar 50 pc 2 * syringe 60 ml | 679.05 |
| Contrast - decufill 50 pc | 142.44 |
| Anesthesia Lump Sum | 168.56 |

Results of Micro Costing Study

**Fig. S1**. Orange line shows the cumulative cost per treatment (in €). Blue bars show the cost per module of the care pathway.

Cumulative Costs per treatment

|  |  |  |
| --- | --- | --- |
| Outpatient Consultation | 156 | 156 |
| Pretreatment Image | 689.59 | 845.59 |
| Preparation | 765.56 | 1611.15 |
| Optimization | 1341.48 | 2952.63 |
| Sonication | 1963.38 | 4916.01 |
| Post Treatment | 531.6 | 5447.61 |
| Recovery | 100.7 | 5548.31 |
| Anesthesia | 420.24 | 5968.55 |

 **Fig. S2.** Costs in scenario analysis.

# Online resource 3 – Model Validation

The validation process of the model is reported below according to the questions of the AdViSHE checklist [[1](bookmark://_ENREF_1)]. which is divided in 4 parts:

**Part A: Validation of the conceptual model (2 questions)**

**A1/ Face validity testing (conceptual model): Have experts been asked to judge the appropriateness of the conceptual model?**

Yes. the conceptual model was checked by all authors.

**A2/ Cross validity testing (conceptual model): Has this model been compared to other conceptual models found in the literature or clinical textbooks?**

To cross-validate the concept of our model. we compared it to other cost-utility analyses published before:

The concept of the model was developed based in similar models comparing different strategies to the treatment of bone metastases. For instance, the health states considered are similar and the transitions are equivalent to other Markov models. The assumption regarding opioid intake (oxycodone) in all states except for complete pain relief was also applied in other models, and the simplification of accounting for all fractures as one homogenous groups was also done by other modelling studies [3, 4].

**Part B: Input data validation (2 questions)**

**B1/ Face validity testing (input data): Have experts been asked to judge the appropriateness of the input data?**

All authors were asked to judge the clinical and effectiveness data.

In this step. the following issues were raised by experts regarding utility values:

- We did not find utility data specific for the Dutch population. We opted to apply a time trade off data because EQ5D might be unable to capture the effect of painful bone metastases. Pain contributes to determining the health status of cancer patients with bone metastases, but the correlation of BPI and EQ5D is low. The single pain item of the EQ-5D may be of limited utility in studies for which pain is an endpoint. The percentages of variation in the EQ-5D utility index scores explained by the BPI pain severity were 28% for non-small cell lung cancer, 30% for multiple myeloma, 31% for breast cancer, 32% for other cancers and 37% for prostate cancer.[5]
- Based on EQ-5D data for patients with chronic pain, for patients with complete and partial pain relief after treatment an increase in utility of 50% and 15%, respectively, was assumed. This assumption was supported by studies showing that less pain predicted for improved QoL, in patients receiving radiation therapy for painful bone metastases[6].
- The assumption that utility gain after MR-HIFU would be equivalent to a response in EBRT was based on studies that show that clinically significant improvements were seen in the QoL scales of physical functioning, fatigue, appetite loss, nausea and vomiting, constipation and pain in the 53% of patients who were classified as responders (traditional criteria) at day 30 after MR-HIFU[7].

In addition, clinical experts considered the comparative study by Napoli et al. from 2023[8] to be more representative of the current MR-HIFU procedures than the randomized clinical trial by Hurwitz et al. from 2014 [9].

Regarding cost data, the following issues were raised:

The assumption that all patients treated with MR-HIFU would be treated with dexamethasone to prevent flare-ups was challenged by one of the centers consulted. Instead, patients might be treated with an increased dosage of analgesics in the one or two days following procedure. However, we opted to keep the assumption, because the expected impact on the model result was expected to be negligible.

After the HIFU procedure, patients are expected to be discharged on the same day. However, it was noted by some experts that some patients might stay overnight. A consensus was not achieved between the two treatment centers consulted during model validation.

**B2/ Model fit testing: When input parameters are based on regression models. have statistical tests been performed?**

We adjusted yearly values to fit the model’s monthly cycles and back transformed rates into probabilities. These calculations were done by JSCG and reviewed by a second model expert (DM).

**Part C: Validation of the computerized model (4 questions)**

**C1/ External review: Has the computerized model been examined by modelling experts?**

Yes. The computerized model was checked by a modelling expert (DM) after data imputation by JSCG. The model trex file was provided for peer review.

**C2/ Extreme value testing: Has the model been run for specific. extreme sets of parameter values in order to detect any coding errors?**

To detect coding errors we tested extreme values for cost data and utility data.

**C3/ Testing of traces: Have patients been tracked through the model to determine whether its logic is correct?**

Not applicable.

**C4/ Unit testing: Have individual sub-modules of the computerized model been tested?**

No sub-models were tested, because the model structure is relatively small with only 3 health states.

**Part D: Operational validation (4 questions)**

**D1/ Face validity testing (model outcomes): Have experts been asked to judge the appropriateness of the model outcomes?**

Yes. The appropriateness of model outcomes was judged by all authors.

**D2/ Cross validation testing (model outcomes): Have the model outcomes been compared to the outcomes of other models that address similar problems?**

The results of our model are generally comparable to that of other modelling studies, as described below.

The potential cost-effectiveness of MR-HIFU in comparison to placebo has been demonstrated in the US setting [3]. In addition, a previous patient-level simulation model showed that MR-HIFU in comparison to EBRT is potentially cost-effective from the payer perspective in Germany, with an incremental cost-effectiveness ratio (ICER) of €19,845/QALY. However, according to national guidance for treatment, in the German model, only 10% of patients in the EBRT group received single fraction, while 90% received multi-fraction [10]. In contrast, in the Netherlands since single fraction is well established as the preferred option when radiotherapy is concerned [11].

**D3/ Validation against outcomes using alternative input data: Have the model outcomes been compared to the outcomes obtained when using alternative input data?**

We tested all variables in DSA. reported in the main manuscript and in the supplementary material.

**D4/ Validation against empirical data: Have the model outcomes been compared to empirical data?**

We validated the mean EQ5D utility values by comparing our mean utility values with prospective data from the Dutch Bone Metastasis Study, a cost-utility based on a randomized trial[11]. In this study mean utility was about 0.4 for the whole follow up period, which is similar to the output of out model.

Regarding pain recurrence rates, a model validation against Dutch cohort lends credibility to our assumption of 20% probability for pain relapse after 1 year. The model input parameters data from the Dutch Bone Trial show that patients who respond to radiotherapy experience pain relief for approximately half of their remaining life with observed reirradiation due to pain relapse after single fraction EBRT of 26% [12].

# Online resource 4 - Calculation of disease severity

The WTP threshold was set by calculating the disease severity index for bone metastases according to the ZIN guideline for health economic evaluations [1]. The reference values for WTP threshold in the Netherlands vary according to burden of disease:

| Burden of disease | Reference value for the maximum additional costs (€) per QALY |
| --- | --- |
| From 0,1 up to 0,4 | €20,000/QALY |
| From 0,41 up to 0,7 | €50,000/QALY |
| From 0,71 up to 1,0 | €80,000/QALY |

We calculated the disease severity index according to both the proportional shortfall and the absolute shortfall as shown below in figure S3. Utility data for patients with bone metastases (0.47) were taken from Matza et al and Svensson et al, as shown in table S5. Results are summarized in table S6.

**Fig. S3.** Calculation of disease severity index. Figure copied from the ZIN guideline [24].

**Table S5**. Input data for the calculation of the disease severity index

|  | Median age at diagnosis (in years) | Median survival time (days) | Median survival time (in years) | Source |
| --- | --- | --- | --- | --- |
| Per primary cancer |  |  |  |  |
| Lung cancer | 66 | 74 | 0.20 | Svensson et al. |
| Breast cancer | 63 | 377 | 0.03 | Svensson et al. |
| Prostate Cancer | 74 | 210 | 0.58 | Svensson et al. |
| General population in the Netherlands | - | - | 82 | OECD index |

**Calculation**

**Lung cancer:**

Remaining QALYs with disease: 0.2 * 0.47 = 0.09

Remaining QALYs without disease (median age lung cancer diagnosis (66)): 82 – 66 = 16

**Breast cancer:**

Remaining QALYs with disease: 1.03 * 0.47 = 0.48

Remaining QALYs without disease (median age breast cancer diagnosis (66)): 82 – 63 = 19

**Prostate cancer:**

Remaining QALYs with disease: 0.58 * 0.47 = 0.27

Remaining QALYs without disease (median age breast cancer diagnosis (66)): 82 – 74 = 8

**Table S6**. Results

| **Cancer type** | **Proportional shortfall** | **Absolute shortfall (Fair innings)** | **WTP Threshold** |
| --- | --- | --- | --- |
| Lung cancer | 0.99 | 15.91 QALYs lost | €80,000/QALY |
| Breast cancer | 0.97 | 18.52 QALYs lost | €80,000/QALY |
| Prostate cancer | 0.97 | 7,72 QALYs lost | €80,000/QALY |

# Online resource 5 – Base case results and subgroup analyses

**Fig. S4.** Base case results plotted in the cost-effectiveness plane

Table S7. Subgroup analyses per cancer type

| Strategy | Cost (€) | Incr Cost (€) | Effectiveness (QALY) | Incr Effectiveness (QALY) | ICER  (€/ QALY) |
| --- | --- | --- | --- | --- | --- |
| Breast Cancer | | | | | |
| SF-EBRT | 6,910 |  | 1.147 |  |  |
| Delayed MR-HIFU | 7,705 | 795 | 1.174 | 0.027 | 29,404 |
| Early MR-HIFU | 8,616 | 1,706 | 1.284 | 0.137 | 12,378 |
| Prostate Cancer | | | | | |
| SF-EBRT | 7,025 |  | 1,242 |  |  |
| Delayed MR-HIFU | 7,829 | 804 | 1,272 | 0.030 | 26,178 |
| Early MR-HIFU | 8,790 | 1,766 | 1,422 | 0.180 | 9,760 |
| Lung Cancer | | | | | |
| SF-EBRT | 5,598 |  | 0,721 |  |  |
| Delayed MR-HIFU | 6,120 | 520 | 0,737 | 0,015 | 32,800 |
| Early MR-HIFU | 7,748 | 2,149 | 0,844 | 0,122 | 17,582 |

# Online resource 6- Scatterplots of probabilistic sensitivity analysis results

(A) Delayed MR-HIFU compared to SF-EBRT.

(B) ‘Early MR-HIFU’ compared to SF-EBRT.

**Fig. S5.** Scatterplots show the results of the probabilistic sensitivity analysis for the two comparisons (A) Delayed MR-HIFU compared to SF-EBRT and (B) ‘Early MR-HIFU’ compared to SF-EBRT. 95% confidence ellipses. Abbreviations: QALY: quality-adjusted life years, WTP: willingness-to-pay threshold.

**References**

1.	Zorginstituut Nederland. *Guideline for economic evaluations in healthcare*. 2024 [cited 2024 12 october]; Available from: <https://english.zorginstituutnederland.nl/publications/reports/2016/06/16/guideline-for-economic-evaluations-in-healthcare>.

2.	De Nederlandse Federatie van Universitair Medische Centra. *Cao University Medical Centres 2024 - 2025*. 2024 [cited 2024 November 1 ]; Available from: <https://www.nfu.nl/sites/default/files/2024-09/Cao_2024-2025_NFU-24.01132_ENG.pdf>.

3.	Bucknor, M.D., et al., *Cost-effectiveness analysis of magnetic resonance-guided focused ultrasound ablation for palliation of refractory painful bone metastases.* Int J Technol Assess Health Care, 2020. **37**: p. e30.

4.	Simões Corrêa Galendi, J., et al., *A time-driven activity-based costing approach of magnetic resonance-guided high-intensity focused ultrasound for cancer-induced bone pain.* Int J Hyperthermia, 2022. **39**(1): p. 173-180.

5.	Mendoza, T.R., et al., *The contribution of pain in determining the health status of cancer patients with bone metastases: A secondary analysis of data from three Phase III registration trials.* Eur J Pain, 2018. **22**(3): p. 565-571.

6.	Westhoff, P.G., et al., *Quality of Life in Relation to Pain Response to Radiation Therapy for Painful Bone Metastases.* Int J Radiat Oncol Biol Phys, 2015. **93**(3): p. 694-701.

7.	Harding, D., et al., *Evaluation of Quality of Life Outcomes Following Palliative Treatment of Bone Metastases with Magnetic Resonance-guided High Intensity Focused Ultrasound: An International Multicentre Study.* Clin Oncol (R Coll Radiol), 2018. **30**(4): p. 233-242.

8.	Napoli, A., et al., *Focused Ultrasound and External Beam Radiation Therapy for Painful Bone Metastases: A Phase II Clinical Trial.* Radiology, 2023. **307**(2): p. e211857.

9.	Hurwitz, M.D., et al., *Magnetic resonance-guided focused ultrasound for patients with painful bone metastases: phase III trial results.* J Natl Cancer Inst, 2014. **106**(5).

10.	Simões Corrêa Galendi, J., et al., *Early economic modeling of magnetic resonance image-guided high intensity focused ultrasound compared to radiotherapy for pain palliation of bone metastases.* Front Oncol, 2022. **12**: p. 987546.

11.	van den Hout, W.B., et al., *Single- versus multiple-fraction radiotherapy in patients with painful bone metastases: cost-utility analysis based on a randomized trial.* J Natl Cancer Inst, 2003. **95**(3): p. 222-9.

12.	Spencer, K., et al., *Net Pain Relief After Palliative Radiation Therapy for Painful Bone Metastases: A Useful Measure to Reflect Response Duration? A Further Analysis of the Dutch Bone Metastasis Study.* Int J Radiat Oncol Biol Phys, 2019. **105**(3): p. 559-566.
